# Case of Gunshot Wound through the Abdomen

**Published:** 1859-08

**Authors:** J. J. Edmunds

**Affiliations:** Buffalo, N. Y.


ART. III.—*Case of Gun-Shot Wound through the Abdomen.—Recovery:* By J. J. Edmunds, M. D., Buffalo, N. Y.

I was called to the Penitentiary of Erie county, on Tuesday morning, March 1st, 1859, to see Peter ———, a colored man and a convict, who, in attempting to escape, had been fired upon by the guard and wounded severely.

I saw him at 9 A. M., with Doct. G. F. Pratt as council, and found him in the following condition, to wit: very much depressed; skin pale; feet and hands cold; pulse slow and feeble. On examination, we found two wounds; one in the centre of the right gluteal region, and the other in the back, nearly opposite the junction of the lumbar with the dorsal vertebræ, about three inches to the left of the spine. The ball had passed through the bowels, wounded the left kidney, and lodged in the abdominal muscles, about two inches above and to the left of the umbilicus.

No attempts were made to extract the balls, and the probe was only used sufficiently to indicate the direction of the wounds. He was ordered to take a draught of brandy immediately—to have morphine, gr. $\frac{1}{4}$, every two hours, with hot bricks and bottles to the extremities. At 11 A. M. he vomited freely—no appearance of reaction. I observed at this time, that the right leg and foot were quite numb, and somewhat colder than the others. The pain was not quite so severe. 4 P. M.: reaction coming on—feet warm—pulse stronger. 6 P. M.: abdominal tenderness and hardness on pressure—pulse full but not hard—urine (drawn by catheter) bloody. The same treatment was continued, with hot fomentations to abdomen.

March 2d, 9 A. M.: Patient cheerful—pulse full and somewhat hard—abdomen tender—urine bloody; same treatment, that is to say, morphine, gr. $\frac{1}{4}$, every two hours—hot fomentations to bowels—crust coffee for drink—no food. 4 P. M.: bears up well—urine not quite so bloody, but presenting evidences of existing inflammation; same treatment.

March 3d, 9 A. M.: No worse—pulse full and about 110 per minute—tongue coated white—some appetite; same treatment continued, with a pint of milk porridge daily. 4 P. M.: About the same—external wounds healing; same treatment.

March 4th, 9 A. M.—Improving—not much fever—no pain—has had no return of the vomiting since the first day—pulse not quite so full—tongue white—appetite increasing; same treatment.

March 5th, 9 A. M.—Better—sweats freely—abdominal tenderness a little more circumscribed. Ordered morphine, gr. $\frac{1}{4}$, every three hours, a mush poultice to cover the bowels, and an extra basin of porridge.

March 6th, 9 A. M.—Patient better—no bad symptoms—bowels have not moved since he was wounded. Ordered morphine, gr. $\frac{1}{4}$, every four hours; other treatment the same.

March 7th, 10 A. M.—Better—pulse slower—bowels have not moved; same treatment.

March 8th.—Patient gaining—bowels have moved freely and spontaneously, three or four times—pulse 88 per minute, and quite natural; same treatment.

March 9th.—Better; complains, however, of soreness about the external wound of the loins, which has a puffy, ulcerative appearance. Ordered morphine, gr. $\frac{1}{4}$, every six hours, and a poultice to the wound over the loins; also a little more food.

March 10th.—Much better—found him sitting up. Complains of lameness and numbness of right leg. Ordered morphine three times a day, with a more liberal allowance of food; the leg to be rubbed with camphor liniment occasionally.

March 13th.—Patient seems nearly well. Complains only of slight lameness; discharged from farther treatment.

*Remarks.* I am not sure that there is anything particularly *unique* or interesting in this case; but a single consideration, has induced me to publish it,—to wit: I have noticed that most surgeons seem to regard a bullet through the intestines, as almost necessarily fatal; and when a case of recovery from such an injury is brought to their notice, they try to explain it, by supposing that the ball either passed round, without entering the peritoneal cavity, or in some manner glided between the intestines, without inflicting injury.

In this case, however, there could be no mistake or room for doubt; the bullet passed directly through the cavity, and the intestine was, without doubt, perforated.

